# The Enigmatic Snow Microorganism, *Chionaster nivalis*, Is Closely Related to *Bartheletia paradoxa* (*Agaricomycotina*, *Basidiomycota*)

**DOI:** 10.1264/jsme2.ME21011

**Published:** 2021-06-16

**Authors:** Ryo Matsuzaki, Yusuke Takashima, Iwane Suzuki, Masanobu Kawachi, Hisayoshi Nozaki, Seiichi Nohara, Yousuke Degawa

**Affiliations:** 1 Faculty of Life and Environmental Sciences, University of Tsukuba, 1–1–1 Tennodai, Tsukuba, Ibaraki, 305–8572, Japan; 2 Biodiversity Division, National Institute for Environmental Studies, 16–2 Onogawa, Tsukuba, Ibaraki, 305–8506, Japan; 3 Sugadaira Research Station, Mountain Science Center, University of Tsukuba, 1278–294 Sugadairakogen, Ueda, Nagano, 386–2204, Japan; 4 Department of Biological Sciences, Graduate School of Science, University of Tokyo, 7–3–1 Hongo, Bunkyo-ku, Tokyo, 113–0033, Japan

**Keywords:** meta-amplicon sequencing data, molecular phylogeny, snow ecosystem, snow fungi

## Abstract

*Chionaster nivalis* is frequently detected in thawing snowpacks and glaciers. However, the taxonomic position of this species above the genus level remains unclear. We herein conducted molecular analyses of *C. nivalis* using the ribosomal RNA operon sequences obtained from more than 200 cells of this species isolated from a field-collected material. Our molecular phylogenetic analyses revealed that *C. nivalis* is a sister to *Bartheletia paradoxa*, which is an orphan basal lineage of *Agaricomycotina*. We also showed that *C. nivalis* sequences were contained in several previously examined meta-amplicon sequence datasets from snowpacks and glaciers in the Northern Hemisphere and Antarctica.

Food webs in thawing snow and glaciers are comprised of cold-adapted microorganisms, such as algae, heterotrophic protists (*e.g.*, ciliates and heterokonts), rotifers, fungi, and bacteria (Hoham and Duval, 2001; Hoham and Remias, 2020). The ecosystems in these harsh environments have long attracted the interest of biologists, resulting in ongoing efforts to elucidate their biodiversity. Recent high-throughput sequencing techniques revealed comprehensive communities within these ecosystems, particularly in microalgae and bacteria (*e.g.*, Lutz *et al.*, 2015, 2016; Segawa *et al.*, 2018; Krug *et al.*, 2020). The outcomes of high-throughput sequencing for the identification and classification of detected microorganisms depend on the quality of the reference databases (Lutz *et al.*, 2019). Therefore, it is important to increase the availability and taxonomic accuracy of molecular data on microorganisms inhabiting snow and glaciers.

*Chionaster nivalis* is one of the typical unicellular microorganisms found within thawing snowpacks and glaciers, occurring in blooms of snow-inhabiting microalgae (*i.e.*, colored snow). This species is characterized by a star-shaped cell wall that is formed by three to five protuberances (Bohlin, 1893; Wille, 1903). Possibly due to its unique morphological traits, this species has frequently been detected in snowpacks and glaciers in Europe, North America, Japan, Australia, and Russia as well as in the Arctic region (Kol, 1968; Marchant, 1982; Müller *et al.*, 1998; Uetake *et al.*, 2006). Therefore, *C. nivalis* is regarded as a widely distributed species. Despite extensive studies, the taxonomic position of *C. nivalis* above the genus level remains unclear. This species was originally described by Bohlin (1893) based on the red snow material collected in Sweden. Although the majority of cells within the material appeared to lose their color due to the preservation solution, Bohlin classified the organism as the third species in the unicellular green algal genus, *Cerasterias* because the cell morphology of this organism was consistent with the concept of the genus being characterized by the presence of several protuberances on cells (Reinsch, 1866; but see also Komárek and Fott, 1983). Wille (1903) subsequently transferred this species to the new fungal genus, *Chionaster*, due to the observation that the organism was completely colorless. However, Wille only reported that this species needed to be listed as a new family and did not state its taxonomic position above the genus level. MycoBank (www.mycobank.org) currently treats *C. nivalis* as Fungi *incertae sedis*, whereas Index Fungorum (http://www.indexfungorum.org/) assigns this species to Chromista *incertae sedis*. There are no molecular data available for this species, possibly due to the lack of cultures of *C. nivalis* and also because this species does not form monospecific blooms in nature (Fukushima, 1963; Kol, 1968).

In our recent field work on melting snowpacks in Japan, we collected several samples of colored snow containing cells that were morphologically identifiable as *C. nivalis* (see Method S1). These cells were not dominant in the samples, as previously described (Fukushima, 1963; Kol, 1968). Viewed under a light microscope, cells were solitary and generally had four protuberances that formed a cross (Fig. 1a). There were sometimes three (Fig. 1b) or five protuberances, and each protuberance was 20–30‍ ‍μm in length with an obtuse apex. The cellular protoplasm was colorless and amorphous in shape, and was generally positioned near the bases of the protuberances. These characteristics were consistent with descriptions of *C. nivalis* from Europe (Bohlin, 1893; Wille, 1903) and Japan (Fukushima, 1963). An attempt was made to establish a culture of Japanese *C. nivalis* by isolating field-collected cells into several conventional culture media for fungi and freshwater and snow-inhabiting microalgae in a cold experimental room at 5°C. However, neither cell division nor spore germination by *C. nivalis* was observed during the study and attempts completely failed. These findings are consistent with previous descriptions of these cells apparently being hypnospores (Bohlin, 1893; Wille, 1903; Fukushima, 1963), suggesting that additional factor(s) for the induction of germination are required to establish cultures of this species. Alternatively, the culture media used in this study were not suitable for *C. nivalis*.

Since *C. nivalis* was uncultured and did not dominate within samples, we attempted to extract total DNA from an isolated single cell or a few (between 6 and 11) cells of field-collected *C. nivalis* in order to sequence the internal transcribed spacer 2 (ITS-2) region by nested PCR (see Method S2). Only three out of 30 PCR products showed amplification (Fig. S1); however, we obtained faint PCR products and their sequences were of low quality (data not shown). This may have been due to the low quantity and quality of DNA extracted from a single to a few cells. Therefore, we isolated more than 200 cells of the species from field-collected materials to obtain a sufficient quantity of total DNA for PCR (see Method S3 for details). To eliminate contaminants, the surface of *C. nivalis* cells within a snow sample was treated with a sterilizing solution (1% [v/v] antiformin containing 0.1% [v/v] Triton X-100 [Kawai-Toyooka *et al.*, 2004]). Using a capillary pipette, approximately 500 and 200 cells of *C. nivalis* were isolated from colored snow samples collected from Oze National Park (Fig. 1c) and Tambara-kogen Plateau (Fig. 1d), Gunma, Japan, respectively. Isolated cells were grouped by locality and assigned specimen labels of Oze (Fig. S2) and Tambara (Fig. S3), respectively. Each specimen was treated on ice with cold-active nuclease (Cryonase; Takara Bio) to eliminate nontarget nucleotides outside of the cells. According to a previously described method by Nakada *et al.* (2007), *C. nivalis* cells in each specimen were broken using ceramic beads to allow total DNA extraction.

Using extracted DNA from the two specimens, we amplified part of the rRNA operon (including 18S rRNA, internal transcribed spacer 1 [ITS-1], 5.8S rRNA, ITS-2, and 28S rRNA) by PCR, and then directly Sanger-sequenced the PCR products (see Method S3). In the PCR of each DNA sample, we used universal primers for eukaryotic rRNA operons (Table S1 and Fig. S4). The 18S rDNA sequences of the Oze and Tambara specimens harbored four and three group I introns, respectively (Fig. S4 and S5). However, the sequences of the 18S and 28S rRNA-coding regions and rapidly evolving ITS-1 and ITS-2 regions were identical between the two specimens, supporting the hypothesis that the obtained sequence of the rRNA operon originated from a single species, *C. nivalis*.

To construct a phylogenetic tree, Bayesian inference (BI) using MrBayes 3.2.7 (Ronquist *et al.*, 2012) and the maximum likelihood (ML) analysis using RAxML-NG 0.9 (Kozlov *et al.*, 2019) were performed, as previously described by Nozaki *et al.* (2010) and Matsuzaki *et al.* (2021), respectively. Phylogenetic analyses based on 18S rRNA (1,610 bp) and 28S rRNA (549 bp) were conducted for comparisons (Fig. S6 and S7). Since robust discrepancies in phylogenetic relationships within the phylum *Basidiomycota* were not detected between the trees, the concatenated 2,159-bp data matrix of 18S and 28S rRNA was subjected to the same methods as those described above (see Method S4 and Table S2 for details).

Several meta-amplicon sequencing datasets of 18S rRNA and ITS-2 from previous studies on snowpacks and glaciers are available in DDBJ/ENA/GenBank (Table S3); therefore, we attempted to detect the sequences assignable to *C. nivalis* in these datasets using Vsearch 2.15.1 (Rognes *et al.*, 2016). Thresholds for identical matches in 18S rRNA and ITS-2 were set as 0.99 and 0.97, respectively (see also Method S5).

Our molecular phylogenetic analyses demonstrated that *C. nivalis* (Oze and Tambara specimens) belongs to the phylum *Basidiomycota* (Fig. 2, S6, and S7). Within *Basidiomycota*, the monophyly of the subphyla *Pucciniomycotina*, *Ustilaginomycotina*, and *Wallemiomycotina* was well recovered (1.00 posterior probabilities [PP] in BI and 100% bootstrap value [BV] [Felsenstein, 1985] in the ML analysis), whereas that of *Agaricomycotina* was low (only 52% BV in the ML analysis). *C. nivalis* was positioned within *Agaricomycotina* and was a sister to *Bartheletia paradoxa* (*Bartheletiomycetes*) with robust statistical support (1.00 PP in BI and 100% BV in the ML analysis). *B. paradoxa* is a filamentous fungus associated with fallen *Ginkgo biloba* leaves, and is known as an orphan basal lineage of *Agaricomycotina* (Mishra *et al.*, 2018). Within *Agaricomycotina*, the monophyly of *Agaricomycetes*, *Dacrymycetes*, and *Tremellomycetes* (*i.e.*, *Agaricomycotina*, excluding *B. paradoxa* and *C. nivalis*) was moderately supported (1.00 PP in BI and 86% BV in the ML analysis).

We detected sequences assignable to *C. nivalis* (or at least its relatives) in meta-amplicon sequencing data from snowpacks and glaciers in Arctic regions, Asia (Japan), Europe, North America, and Antarctica (Table 1). To the best of our knowledge, this is the first study on *C. nivalis* from Antarctica; however, the evidence obtained is based solely on molecular data. The present results strongly support the hypothesis that *C. nivalis* is a widely distributed or cosmopolitan species. The ratios of the *C. nivalis* sequences detected in respective sequencing data for each sampling locality were very low (generally less than 1%), which may have been due to the difficulties associated with disrupting the thick cell wall during DNA extraction (see also Fig. S1). Sequence similarities in ITS-2 (known as a highly evolving region) between Japanese *C. nivalis* and sequences from the Northern Hemisphere were as high as 99.4%–100%, whereas those from Antarctica had similarities of at most 97.1%. Although it is important to note that available data were highly limited and further studies are warranted, these sequence similarities indicate that *C. nivalis* diversified between Antarctica and the other regions examined (all of which are in the Northern Hemisphere). Potentially similar cases were reported in the snow-inhabiting microalga, *Sanguina nivaloides* (formerly known as the cyst of *Chlamydomonas nivalis*) (Segawa *et al.*, 2018; Brown and Tucker, 2020).

In the present study, we collected hundreds of *C. nivalis* cells from a single source of field material, and performed PCR and Sanger sequencing using DNA extracted from these cells. This method may be applicable to molecular analyses of microorganisms that are not culturable and do not form monospecific blooms in nature. Our molecular data clearly demonstrated that *C. nivalis* is closely related to *B. paradoxa*, known as the basal lineage of *Agaricomycotina*. The former species (*C. nivalis*) has only been reported from snow and glacier environments, whereas the latter (*B. paradoxa*) grows only on fallen leaves of *G. biloba*. Since the morphological, ecological, and life cycle data of *C. nivalis* are highly limited, further discussions, including the taxonomic position of the species at the class level (*Bartheletiomycetes* or a new class altogether), are difficult. However, the present results provide insights into this enigmatic snow fungus and the basal lineage of *Agaricomycotina*. In addition, we demonstrated that the sequences assignable to *C. nivalis* were actually present in several meta-amplicon sequencing datasets from previously examined snowpacks and glaciers. This strongly supports previous findings by Lutz *et al.* (2019) indicating the importance of enlarging reference databases with accurate taxonomic information to reveal whole microbial communities in field-collected materials using high-throughput sequencing technologies.

## Data availability

All sequence data obtained in the present study have been deposited in DDBJ/ENA/GenBank under the accession numbers LC599386–7, MW404634–6, and MW404638–40. The sequence matrix used in the present study is available from TreeBASE (www.treebase.org/treebase-web/home.html, study ID: S27454).

## Citation

Matsuzaki, R., Takashima, Y., Suzuki, I., Kawachi, M., Nozaki, H., Nohara, S., and Degawa, Y. (2021) The Enigmatic Snow Microorganism, *Chionaster nivalis*, Is Closely Related to *Bartheletia paradoxa* (*Agaricomycotina*, *Basidiomycota*). *Microbes Environ ***36**: ME21011.

https://doi.org/10.1264/jsme2.ME21011

## Supplementary Material

Supplementary Material 1

Supplementary Material 2

## Figures and Tables

**Fig. 1.**
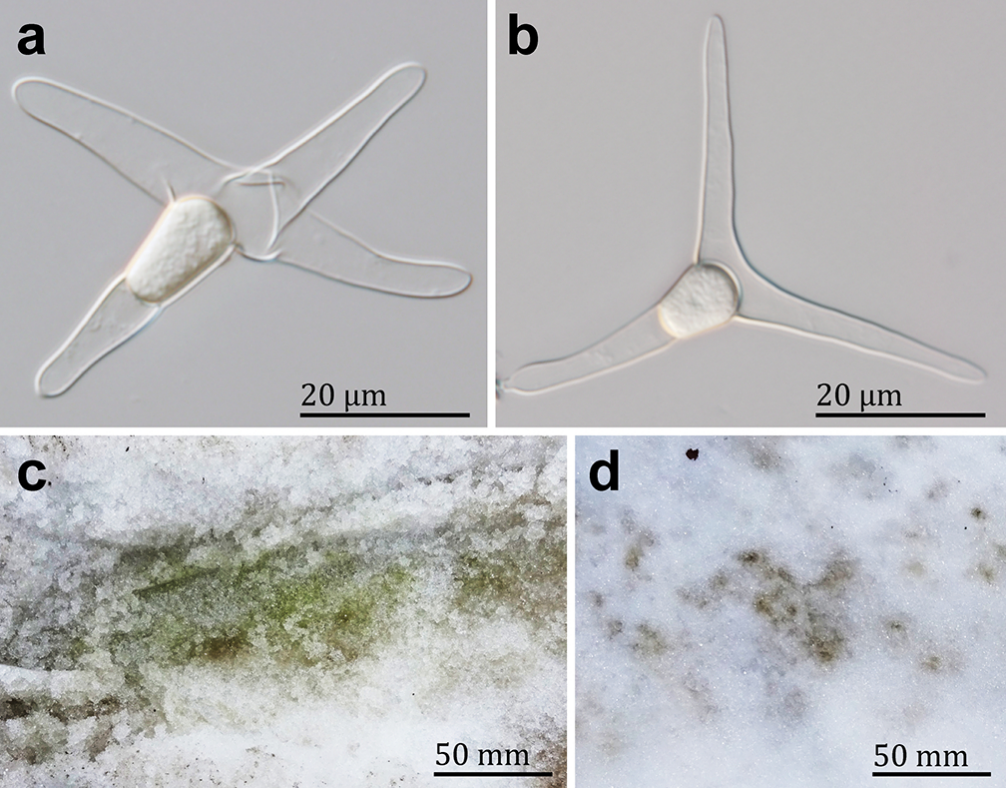
*Chionaster nivalis* from Japan. (a, b) Light micrographs of *C. nivalis* cells from Japan with four (a) and three (b) protuberances. (c, d) Colored snow materials used in the present study. (c) Green snow near coniferous trees, Oze National Park, Gunma, Japan (36°55′16.68″ N, 139°18′39.53″ E; elevation 1,651 m) on May 2, 2019. (d) Brown snow under naked broad-leaved trees, Tambara-kogen Plateau, Gunma, Japan (36°47'13.04" N, 139°03'26.68" E; elevation 1,237 m) on April 28, 2019.

**Fig. 2.**
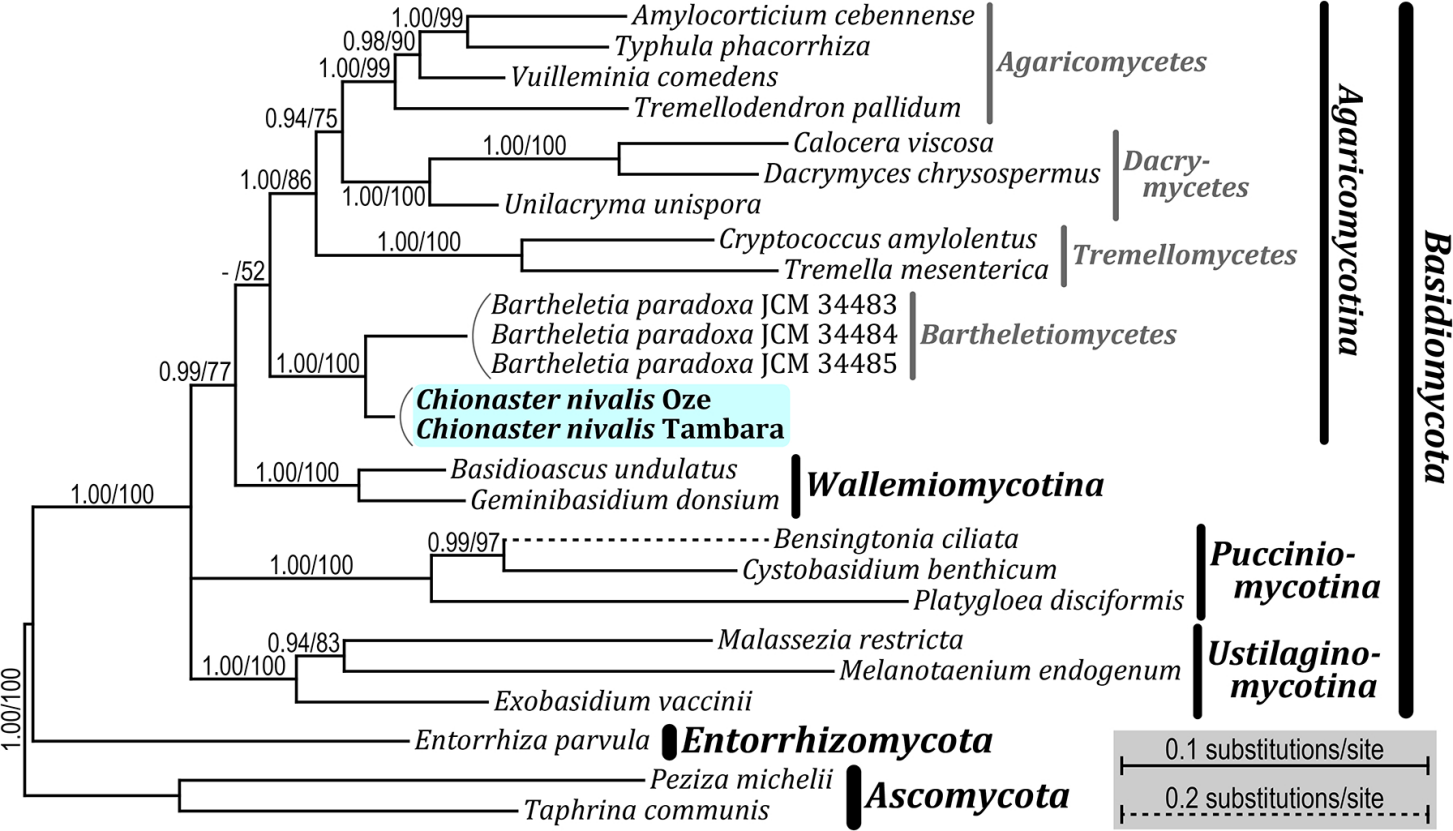
Bayesian phylogenetic tree of *Basidiomycota* based on 18S and 28S ribosomal RNA sequences. Names of classes, subphyla, and phyla are according to the previous study by Naranjo-Ortiz and Gabaldón (2019). The corresponding posterior probabilities of Bayesian inference (0.90 or more, left) and bootstrap values from a maximum likelihood analysis (50% or more, right) are shown at each node.

**Table 1.** Meta-amplicon sequencing data from snowpacks and glaciers containing sequences assignable to *Chionaster nivalis*. See Table S3 for detailed information.

Region	Country	Study	Molecular marker	% of identical matches^1^	% of detected reads in each sequencing
Arctic	Norway (Svalbard) or Sweden^2^	Lutz *et al.*, 2017	18S rRNA	99.1–100	0.002
	Norway (Svalbard)	Lutz *et al.*, 2016	18S rRNA	99.1–99.4	0.02–0.03
		Segawa *et al.*, 2018	ITS-2	97.1–99.6	0.01–1.6
Asia	Japan	Terashima *et al.*, 2017	18S rRNA	99.1–100	0.004–0.8
Europe	Austria	Lutz *et al.*, 2019	18S rRNA	99.1–100	0.01–0.2
			ITS-2	97.1–100	0.01–0.8
	Denmark (Greenland)	Segawa *et al.*, 2018	ITS-2	97.1–100	0.007–0.03
North America	USA^2^	Brown *et al.*, 2015	ITS-2	97.1–100	0.4
		Segawa *et al.*, 2018	ITS-2	97.1–100	0.001–0.9
	Canada	Yakimovich *et al.*, 2020	18S rRNA	99.1–100	0.0003–0.08
Antarctic	(Antarctica)	Segawa *et al.*, 2018	ITS-2	97.1	0.001–0.01

^1^ Thresholds are >0.99 in 18S rRNA and >0.97 in the ITS-2 region. ^2^ Available data are not subdivided into the respective areas.
