# Supplementary material for: The Enigmatic Snow Microorganism, Chionaster nivalis, Is Closely Related to Bartheletia paradoxa (Agaricomycotina, Basidiomycota)
Source: Microbes Environ. 2021 Jun 16;36(2):ME21011. doi: 10.1264/jsme2.ME21011 (PMC8209449; doi:10.1264/jsme2.ME21011)
Supplement: Supplementary file 1 — Supplementary Material 1 [file 36_21011_s1.pdf]

Supplementary Materials
The Enigmatic Snow Microorganism, *Chionaster nivalis*, Is Closely Related to *Bartheletia paradoxa* (*Agaricomycotina*, *Basidiomycota*)

RYO MATSUZAKI[1,2]*, YUSUKE TAKASHIMA[1,3], IWANE SUZUKI[1], MASANOBU KAWACHI[2], HISAYOSHI NOZAKI[4], SEIICHI NOHARA[2], and YOUSUKE DEGAWA[1,3]

[1]*Faculty of Life and Environmental Sciences, University of Tsukuba, 1-1-1 Tennodai, Tsukuba, Ibaraki, 305-8572, Japan*
[2]*Biodiversity Division, National Institute for Environmental Studies, 16-2 Onogawa, Tsukuba, Ibaraki, 305-8506, Japan*
[3]*Sugadaira Research Station, Mountain Science Center, University of Tsukuba, 1278-294 Sugadairakogen, Ueda, Nagano, 386-2204, Japan*
[4]*Department of Biological Sciences, Graduate School of Science, University of Tokyo, 7-3-1 Hongo, Bunkyo-ku, Tokyo, 113-0033, Japan*

*Corresponding Author: matsuzaki.ryo@nies.go.jp

This file contains:

Method S1. Collection and light microscopy of field materials.
Method S2. Single-cell- and few-cell-sequencing for Japanese *Chionaster nivalis*.
Method S3. DNA extraction, PCR, and sequencing for Japanese *Chionaster nivalis* and *Bartheletia paradoxa*.
Method S4. Molecular phylogenetic analyses.
Method S5. Comparison with meta-amplicon sequencing data in the previous studies.

Table S1. Primers used for amplification and sequencing of nuclear ribosomal RNA operon.
Table S2. Taxa/specimens/strains in the present phylogenetic analyses and their DDBJ/ENA/GenBank accession numbers of 18S and 28S ribosomal RNA.
(**Table S3** is provided as a distinct Excel file.)

Fig. S1. Results of the secondary PCR for the DNA extracted from a single cell or few cells of *Chionaster nivalis*.
Fig. S2. Isolated *ca.* 500 cells of *Chionaster nivalis* from the green snow sample of Oze National Park (Fig. 1c), shown as a composite micrograph.
Fig. S3. Isolated *ca.* 200 cells of *Chionaster nivalis* from the brown snow sample of Tambara-kogen Plateau (Fig. 1d), shown as a composite micrograph.
Fig. S4. Schematic diagram of the Sanger-sequenced nuclear ribosomal RNA operon of *Chionaster nivalis* specimens Oze and Tambara.
Fig. S5. Results of the PCR for the DNA samples obtained from more than 200 cells of *Chionaster nivalis*.
Fig. S6. Bayesian phylogenetic tree of *Basidiomycota* based on 18S ribosomal RNA sequences.
Fig. S7. Bayesian phylogenetic tree of *Basidiomycota* based on 28S ribosomal RNA sequences.

Supplementary Methods

Method S1. Collection and light microscopy of field materials.

Colored snow samples containing cells morphologically identifiable to *Chionaster nivalis* were collected from snowpacks in two places in Gunma Prefecture, Japan. In Oze National Park, no specific permission was required because collecting snowpacks containing protists from national parks is not legally restricted in Japan. In addition, we confirmed that the samples did not contain protected organisms. The fieldwork in Tambara-Kogen Plateau was conducted with permission from the Tone-Numata District Forest Office. Methods for collecting snow samples and preserving them in the laboratory were the same as those described by Muramoto *et al.* (2010). Light microscopy of *C. nivalis* was carried out at room temperature under an Olympus BX51 light microscope equipped with Nomarski differential interference optics and an Olympus DP72 digital camera (Olympus, Tokyo, Japan). An attempt to establish a culture of Japanese *C. nivalis* was conducted in a cold experimental room at 5°C using the pipette-washing method (Pringsheim, 1946) with several culture media for freshwater and snow-inhabiting microalgae [AF-6 (Kato, 1982, modified according to Kasai *et al.*, 2009); AF-6/3 (Nozaki *et al.*, 2015); SVM (Kirk and Kirk, 1983) modified by 200 mg L^{-1} of sodium acetate (Sugasawa *et al.*, 2015); and mAC (Nozaki *et al.*, 1995)] and autoclaved meltwater from the field-collected snow. In addition, other conventional culture media for fungi [LCA (Miura and Kudo, 1970) and Potato dextrose agar, Corn meal agar, and Malt extract agar (Nissui Pharmaceutical, Tokyo, Japan)] on which *Bartheletia paradoxa* can grow, were also adopted. However, neither cell division nor spore germination of *C. nivalis* was observed.

For obtaining gene sequences of 18S and 28S rRNA, three Japanese strains of *B. paradoxa* were established from a single basidiospore (strains NN46:196-1 and NN46:196-3) and conidium (strain NN46:196-2), collected from two places in Nagano Prefecture, Japan (36°24'17.69" N, 138°14'40.55" E for NN46:196-1 and NN46:196-2, and 36°27'02.60" N, 138°19'52.10" E for NN46:196-3) on November 18, 2020. The strains NN46:196-1, NN46:196-2, and NN46:196-3 were deposited in the Japan Collection of Microorganisms (JCM) as JCM 34483, JCM 34484, and JCM 34485, respectively.

Method S2. Single-cell- and few-cell-sequencing for Japanese *Chionaster nivalis*.

Chionaster nivalis cells within a snow sample from Oze National Park were treated with a sterilizing solution [1% (v/v) antiformin containing 0.1% (v/v) Triton X-100 (Kawai-Toyooka *et al.*, 2004)], then the cells were rinsed with sterilized Milli-Q water (EMD Millipore, MA, USA). Using a capillary pipette and an Olympus CK40 microscope (Olympus) equipped with a Leica MC170 HD digital camera (Leica Microsystems GmbH, Wetzlar, Germany), a single cell or few (six to 11) cells morphologically identifiable to *C. nivalis* were isolated from the sample at room temperature.

The isolated cells were subdivided into two groups and subjected to different method for DNA extraction. For one group, each isolated cell(s) was suspended in 60 µl 2×PCR Buffer of KOD FX Neo (Toyobo, Osaka, Japan) and pipetted into a 2-ml tube with lysing matrix A, provided by FastDNA SPIN Kit (MP Biomedicals, CA, USA).

After shaking the tube at 25 rotations·s^{-1} for 3 min using a Mini Bead Beater-1 (Biospec Products, OK, USA) and brief centrifugation, collectable 16.5 µl 2×PCR Buffer of KOD FX Neo was used as a template for PCR. For the other group, each isolated cell(s) was suspended in 3.5 µl Lyse-and-Go PCR Reagent (Thermo Fisher Scientific, MA, USA) in a single PCR tube. To lyse the cell, the mixture was heated according to the manufacture's recommendation as follows: 65°C for 30 s; 8°C for 30 s; 65°C for 90 s; 97°C for 180 s; 8°C for 60s; 65°C for 180 s; 97°C for 60 s; 65°C for 60 s. The mixture was then kept at 80°C and used as a template for PCR.

The initial PCR was performed using the primers ITS-Fa and 28S-NL4 (Table S1) and KOD FX Neo (Toyobo) for the former method (with lysing matrix A) or KOD One PCR Master Mix (Toyobo) for the latter method (with Lyse-and-Go PCR reagent), according to the manufactures' protocols with 35 cycles. The PCR products were immediately purified using a FastGene Gel/PCR Extraction Kit (Nippon Genetics, Tokyo, Japan), and were then used for secondary PCR. The secondary PCR was carried out using KOD One PCR Master Mix as described above, with the primers ITS-Fc and ITS-Rb (Table S1).

After confirming the amplification by gel electrophoresis (Fig. S1), the secondary-PCR products were purified as described above and used for a cycle sequence reaction with a BigDye Terminator v3.1 Cycle Sequencing Kit (Thermo Fisher Scientific) based on the manufacturers' instructions. The cycle sequencing products were purified using a BigDye Xterminator Purification Kit (Thermo Fisher Scientific), and electrophoresis was carried out using an Applied Biosystems 3730xl DNA Analyzer (Thermo Fisher Scientific).

Since the sequences obtained by this method were of low quality (data not shown), we did not use the data in this study.

Method S3. DNA extraction, PCR, and sequencing for Japanese *Chionaster nivalis* and *Bartheletia paradoxa*.

Chionaster nivalis cells within a snow sample were sterilized and rinsed as described in Method S2. Using a capillary pipette and an Olympus CK40 microscope (Olympus) equipped with a Leica MC170 HD digital camera (Leica Microsystems GmbH), approximately 500 and 200 cells morphologically identifiable to *C. nivalis* were isolated from the samples originating from Oze National Park and Tambara-kogen Plateau, respectively, at room temperature, and assigned to the specimens Oze (Fig. S2) and Tambara (Fig. S3). For each specimen, the isolated cells were suspended in 5 µl of sterilized MilliQ-water in a 2.0-ml graduated microcentrifuge tube and were treated with 0.5 µl (10 units) of cold-active nuclease (Cryonase; Takara Bio Inc., Shiga, Japan) and 5 µl of 25 mM $MgCl_2$ for 30 min on ice, to eliminate nontarget nucleotides outside of the cells. Subsequently, the mixture containing the cells was subjected to DNA extraction.

The total DNA extraction method used for the specimens of isolated *C. nivalis* cells (Fig. S2 and S3) was essentially the same as that described by Nakada *et al.* (2007). The sterilized cells were resuspended in 300 µl of the extraction buffer [containing 1M NaCl, 0.07M Tris-HCl (pH 8.0), and 0.03M EDTA (pH 8.0)], 25 µl of 10% CTAB solution with 0.7M NaCl, and 300 µl of chloroform, and were shaken with ceramic beads at 25

rotations·s^{-1} for 5 min using a Mini Bead Beater-1 (Biospec Products). After centrifugation by 20,000 × *g* for 2 min, total DNA in the supernatant was extracted using an illustra blood genomicPrep Mini Spin Kit (GE Healthcare UK, Buckinghamshire, UK) according to the manufacturer's instructions. A part of the nuclear rRNA operon [containing 18S rRNA, internal transcribed spacer 1 (ITS-1), 5.8S rRNA, internal transcribed spacer 2 (ITS-2), and 28S rRNA] of each specimen was amplified using KOD FX Neo or KOD One PCR Master Mix (Toyobo) with the five pairs of universal primers for eukaryotic rRNA operons, 18S-FA–18S-RB, 18S-FA–ITS-Rb, ITS-Fa–ITS-Rb, ITS-Fa–28S-NL4, and 28S-NL1–28S-NL4 (Table S1 and Fig. S4 and S5). The PCR mixture composition and PCR conditions were prepared according to the manufacturer's instructions. The purification of PCR products, cycle sequence reaction, purification of the cycle sequencing products, and electrophoresis were performed as described in Method S2. For the cycle sequencing reactions, the primers 18S-FC, 18S-RD, 18S-FE, 18S-RF, 18Sint2-F2, 18Sint2-R3, 18Sint2-R4, 18Sint3-F2, 18Sint3-F3, 18Sint3-R4, ITS-Fc, and ITS-Rd (Table S1) were used, in addition to those used for PCR. The obtained sequences were manually assembled using BioEdit version 7.2.5 (Hall, 1999). Group I introns inserted in the 18S rRNA gene (Fig. S4) were detected based on comparison with the 18S rRNA sequence from *Mrakia frigida* (DDBJ/ENA/GenBank accession number DQ831017). The direct sequencing methodology for the DNA samples showed unambiguous data, except for three and six nucleotides in the Oze and Tambara specimens, respectively, positioned within the group I introns in the 18S rRNA gene. Therefore, we did not clone the PCR products.

For *Bartheletia paradoxa*, template DNA was isolated from pure cultures originating from Japan (JCM 34483–JCM 34485; see Method S1), using Prepman Ultra sample reagent (Thermo Fisher Scientific). The PCR amplifications were performed using KOD FX Neo DNA polymerase (Toyobo) with the fungal universal primers NS1–NS8 and ITS5–LR5 for the 18S and 28S rRNA genes of each culture, respectively (Table S1). The PCR mixture composition and PCR conditions were the same as those described by Takashima *et al.* (2018), with the exception of the annealing temperature (50°C and 54°C for the 18S and 28S rRNA gene amplifications, respectively). The PCR products were purified using polyethylene glycol and ethanol precipitation, and a cycle sequence reaction was performed as described above. The primers used for the cycle sequencing reactions were NS1, NS3, NS4, NS5, NS6, NS7, and NS8 (18S rRNA), and LR0R and LR5 (28S rRNA; see Table S1). The cycle sequencing products were purified by ethanol precipitation, and electrophoresis was performed using an Applied Biosystems 3130xl Genetic Analyzer (Thermo Fisher Scientific) or the DNA sequencing service by FASMAC Co., Ltd. (Kanagawa, Japan). The sequences obtained from each primer were assembled into a single sequence using GeneStudio Professional software version 2.2.0.0 (www.genestudio.com). The obtained sequences of the 18S and 28S rRNA genes were identical among the three Japanese strains of *B. paradoxa*. The sequence similarities between the present Japanese strains and the previously examined specimen from Czech Republic (PRC 3336; Koukol and Lotz-Winter, 2016) were 100% (949/949 bp, vs. LT560339) and 99.82% (541/542 bp, vs. LT560340) in the 18S and 28S rRNA genes, respectively.

Method S4. Molecular phylogenetic analyses.

We used 20 operational taxonomic units (OTUs) of *Basidiomycota*, two OTUs of *Ascomycota*, and one OTU of *Entorrhizomycota* in addition to the two Japanese specimens of *C. nivalis* (Oze and Tambara) for our molecular phylogenetic analyses (see Table S2). This is because our preliminary blastn result using 18S rRNA, ITS-2, and 28S rRNA sequences of the Japanese *C. nivalis* as queries indicated that the species is related to fungi in the phylum *Basidiomycota*, including *B. paradoxa*. Since the available 18S rRNA sequence of *B. paradoxa* specimen PRC 3336 from Czech Republic (DDBJ/ENA/GenBank accession number LT560339) is short (949 bp), we obtained 18S and 28S rRNA sequences from three Japanese *B. paradoxa* strains (see Methods S1 and S3). The blastn results also showed that the three environmental sequences in DDBJ/ENA/GenBank from the Russian glacier ice core and Arctic and Alaskan soil samples (AB474395, KR266147, and KF617467, respectively) are related to *C. nivalis*, with 86.81%–92.65% sequence similarities in ITS-2. Since their 18S rRNA sequences are too short, we did not include them in the subsequent phylogenetic analyses. The phyla *Ascomycota* and *Entorrhizomycota* were treated as outgroups according to previous phylogenetic results (Millanes *et al.*, 2011; Zhao *et al.*, 2017). The 18S and 28S rRNA sequences from the examined OTUs were aligned using MAFFT 7.471 (Katoh and Standley, 2013) with the L-INS-i method. After trimming incomplete 5' and 3' regions of the respective 18S and 28S rRNA sequence matrices by hand, ambiguous sites of the sequence matrices were eliminated using trimAl 1.2 (Capella-Gutiérrez *et al.*, 2009) with the "gappyout" option. Identical sequences in each matrix were reduced to a single OTU. The concatenated sequence matrix was deposited in and is available from TreeBASE (www.treebase.org/treebase-web/home.html; study ID: S27454).

To construct a phylogenetic tree, Bayesian inference (BI) was performed for the 1,610-bp data matrix of 18S rRNA and the 549-bp data matrix of 28S rRNA using MrBayes 3.2.7 (Ronquist *et al.*, 2012), respectively, as described previously by Nozaki *et al.* (2010). The appropriate substitution models for 18S and 28S rRNA were selected based on the Bayesian information criterion in ModelTest-NG (Darriba *et al.*, 2020) with the "-T mrbayes" option. The applied models were GTR+I+G4 and GTR+I+G4 for 18S and 28S rRNA, respectively. One million generations of Markov chain Monte Carlo iterations were conducted, and the first 25% were discarded as burn-in. The average standard deviation of split frequencies was below 0.01, indicating convergence of the analysis. In addition, the maximum likelihood analysis using RAxML-NG 0.9 (Kozlov *et al.*, 2019) was also carried out to estimate the bootstrap values (Felsenstein, 1985) based on 1,000 replications. Since robust discrepancies in the phylogenetic relationships within *Basidiomycota* were not detected between the trees (Fig. S6 and S7), the concatenated 2,159-bp data matrix of 18S and 28S rRNA was subjected to the same methods as described above.

Method S5. Comparison with meta-amplicon sequencing data in the previous studies.

To detect the sequences assignable to *C. nivalis*, we obtained several meta-amplicon sequencing datasets from snowpacks and glaciers examined in previous studies (see

Table S3) from the European Nucleotide Archive (https://www.ebi.ac.uk/ena/browser/home). In several data obtained using the Illumina sequencing platforms, only raw reads were available. Thus, the raw read data were operated as follows for comparison. The adapter sequences of the forward and reverse reads were thoroughly removed using Trim Galore 0.6.6 (Babraham Bioinformatics; http://www.bioinformatics.babraham.ac.uk/projects/trim_galore/). Then, forward and reverse reads were merged using Vsearch 2.15.1 (Rognes *et al.*, 2016) with the default setting of the fastq_mergepairs option. In each dataset, *C. nivalis* sequences were searched using Vsearch with the following conditions: identity > 0.99 in 18S rRNA and > 0.97 in ITS-2, and query and reference coverage > 95%.

Supplementary Tables

Table S1. Primers used for amplification and sequencing of nuclear ribosomal RNA operon.

Designation	Position[1]	Sequence (5'–3')
18S-FA[2]	1–21	AACCTGGTTGATCCTGCCAGT
18S-FC[3]	458–478	GGGAGGTAGTGACAAIAAATA
18S-RD[2]	570–550[4]	GCTGGCACCAGACTTGCCCTC
18S-FE[2]	1111–1131	GGGAGTATGGTCGCAAGGCTG
18S-RF[2]	1201–1181[4]	CCCGTGTTGAGTCAAATTAAG
18Sint2-F2[5]	914–940	TGGATTTACGGAAGACTAACTTCTGCG
18Sint2-R3[5]	252–223[4,6]	ACCCATTGCCATCTAGTCTGTGAACTGCAC
18Sint2-R4[5]	1025–1001[4]	CTGTTAAGACTACAACGGTATCTGC
18Sint3-F2[5]	121–148[7]	TAGAGTCCCAAGCTACCAAGCTGATGTC
18Sint3-F3[5]	241–271[7]	GCACTGGAGATGGTTCAGAGACTAGATGGTC
18Sint3-R4[5]	1232–1206[4]	ATCCTTACTATGTCTGGACCTGGTGAG
NS1[8]	20–38	GTAGTCATATGCTTGTCTC
NS3[8]	554–574	GCAAGTCTGGTGCCAGCAGCC
NS4[8]	1154–1135[4]	CTTCCGTCAATTCCTTTAAG
NS5[8]	1133–1154	AACTTAAAGGAATTGACGGAAG
NS6[8]	1441–1418[4]	GCATCACAGACCTGTTATTGCCTC
NS7[8]	1418–1441	GAGGCAATAACAGGTCTGTGATGC
NS8[8]	1795–1776[4]	TCCGCAGGTTCACCTACGGA
ITS5[8]	1752–1773[10]	GGAAGTAAAAGTCGTAACAAGG
ITS-Fa[9]	1766–1792[10]	GGGATCCGTTTCCGTAGGTGAACCTGC
ITS-Fc[9]	31–50[11]	GCATCGATGAAGAACGCAGC
ITS-Rd[9]	50–33[4,11]	GCTGCGTTCTTCAGCGAT
ITS-Rb[9]	50–23[4,12]	GGGATCCATATGCTTAAGTTCAGCGGGT
28S-NL1[13]	38–60	GCATATCAATAAGCGGAGGAAAG
28S-NL4[13]	693–675[4]	GGTCCGTGTTTCAAGACGG
LR0R[14]	23–39	ACCCGCTGAACTTAAGC
LR5[15]	1002–986[4]	ATCCTGAGGGAAACTTC

Abbreviation: ITS, internal transcribed spacer.

[1]Coodinate numbers from 18S rRNA of *Cryptococcus neoformans* (Fan *et al.*, 1994), 5.8S or 28S rRNA of *Amylocorticium cebennense* (Binder *et al.*, 2010), or second or third intron of 18S rDNA of the *Chionaster nivalis* specimen Oze.

[2]Sourced from Nakazawa and Nozaki (2004).

[3]Sourced from Nakada *et al.* (2007).

[4]Reverse primer.

[5]Designed in this study.

[6]Position in the second intron of 18S rDNA of the *C. nivalis* specimen Oze.

[7]Position in the third intron of 18S rDNA of the *C. nivalis* specimen Oze.

[8]Sourced from White *et al.* (1990).

[9]Sourced from Coleman *et al.* (1994).

[10]Position in 18S rDNA.

[11]Position in 5.8S rDNA.

[12]Position in 28S rDNA.
[13]Sourced from (Kurtzman and Robnett, 1997).
[14]Sourced from the website of Vilgalys Mycology Lab (https://sites.duke.edu/vilgalyslab/rdna_primers_for_fungi/) [accessed 4 January 2021].
[15]Sourced from Vilgalys and Hester (1990).

Table S2. Taxa/specimens/strains in the present phylogenetic analyses and their DDBJ/ENA/GenBank accession numbers of 18S and 28S ribosomal RNA.

		Accession number	
**Taxon**	**Specimen/strain**	**18S rRNA**	**28S rRNA**
Basidiomycota			
**-*Agaricomycotina***			
Amylocorticium cebennense	HHB-2808	GU187612	GU187561
Bartheletia paradoxa	JCM 34483 (= NN46:196-1)	MW404638[1]	MW404634[1]
	JCM 34484 (= NN46:196-2)	MW404639[1]	MW404635[1]
	JCM 34485 (= NN46:196-3)	MW404640[1]	MW404636[1]
Calocera viscosa	TUFC12873	AB712481	AB299048
Chionaster nivalis	Oze	LC599386[1]	LC599386[1]
	Tambara	LC599387[1]	LC599387[1]
Cryptococcus amylolentus	CBS 6039	D64121	AF177404
Dacrymyces chrysospermus	TUFC 13115	AB712494	AB299073
Tremella mesenterica	CBS 6973	NG_061160	NG_069419
Tremellodendron pallidum	AFTOL-ID 699	AY766081	AY745701
Typhula phacorrhiza	IO.14.167(S)	MT232506	MT232315
Unilacryma unispora	TUFC 13839	AB712511	AB712432
Vuilleminia comedens	AFTOL-ID 1247 (= T-583)	AF518594	AF518666
**-*Pucciniomycotina***			
Bensingtonia ciliata	JCM 6865 (= CBS 7514)	D38233	NG_042363
Cystobasidium benthicum	JCM 10901 (= CBS 9124)	AB126647	NG_059003
Platygloea disciformis	IFO 32431	NG_064899	NG_060066
**-*Ustilaginomycotina***			
Exobasidium vaccinii	DSM 5498	MH047198	MH047198
Malassezia restricta	CBS 7877	CP033152	AJ249950
Melanotaenium endogenum	AFTOL-ID 1918	DQ789980	DQ789979
**-*Wallemiomycotina***			
Basidioascus undulatus	DAOM 241956	NG_070805	NG_042692
Geminibasidium donsium	DAOM 241966	NG_061140	NG_042694
***Entorrhizomycota* (outgroup)**			
Entorrhiza parvula	TUB 021488	KM359779	KM359780
***Ascomycota* (outgroup)**			
Taphrina communis	CBS 352.35	NG_065466	MH867214
Peziza michelii	TL 5692	DQ646545	AY500549

[1]Sequenced in this study.

Table S3 (list of meta-amplicon sequencing data examined in this study) is provided as a distinct Excel file.

Supplementary Figures

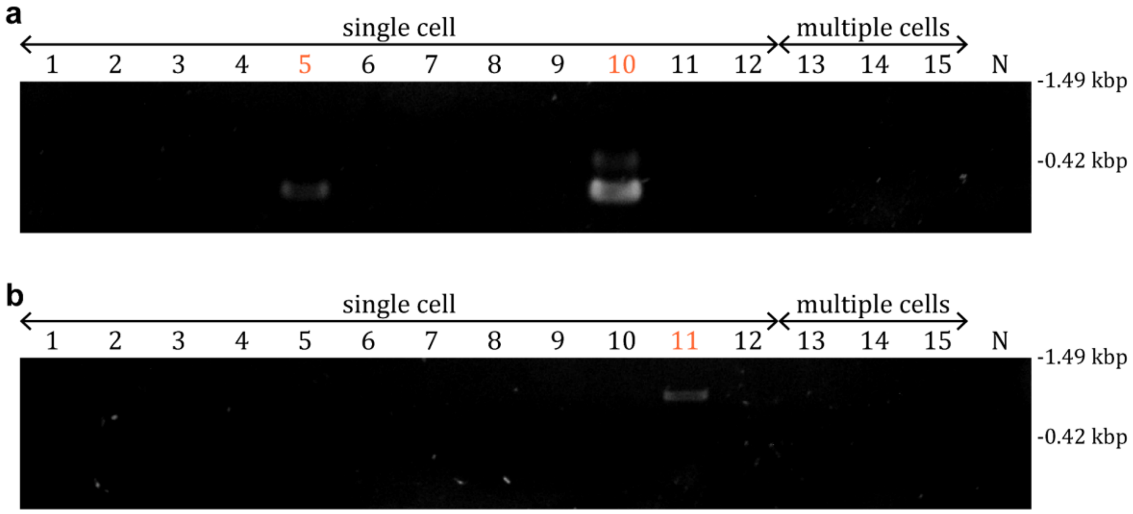

Fig. S1. Results of the secondary PCR for the DNA extracted from a single cell or few cells of *Chionaster nivalis*. (a) DNA samples extracted by a lysing matrix. (b) DNA samples extracted by Lyse-and-Go PCR Reagent. For details, see Method S2. N, negative control. The amplification was detected in the lanes 5 and 10 in (a) and the lane 11 in (b) (shown as orange characters); however, the sequences from the respective PCR products were of low quality (data not shown) and could not be used for further investigation.

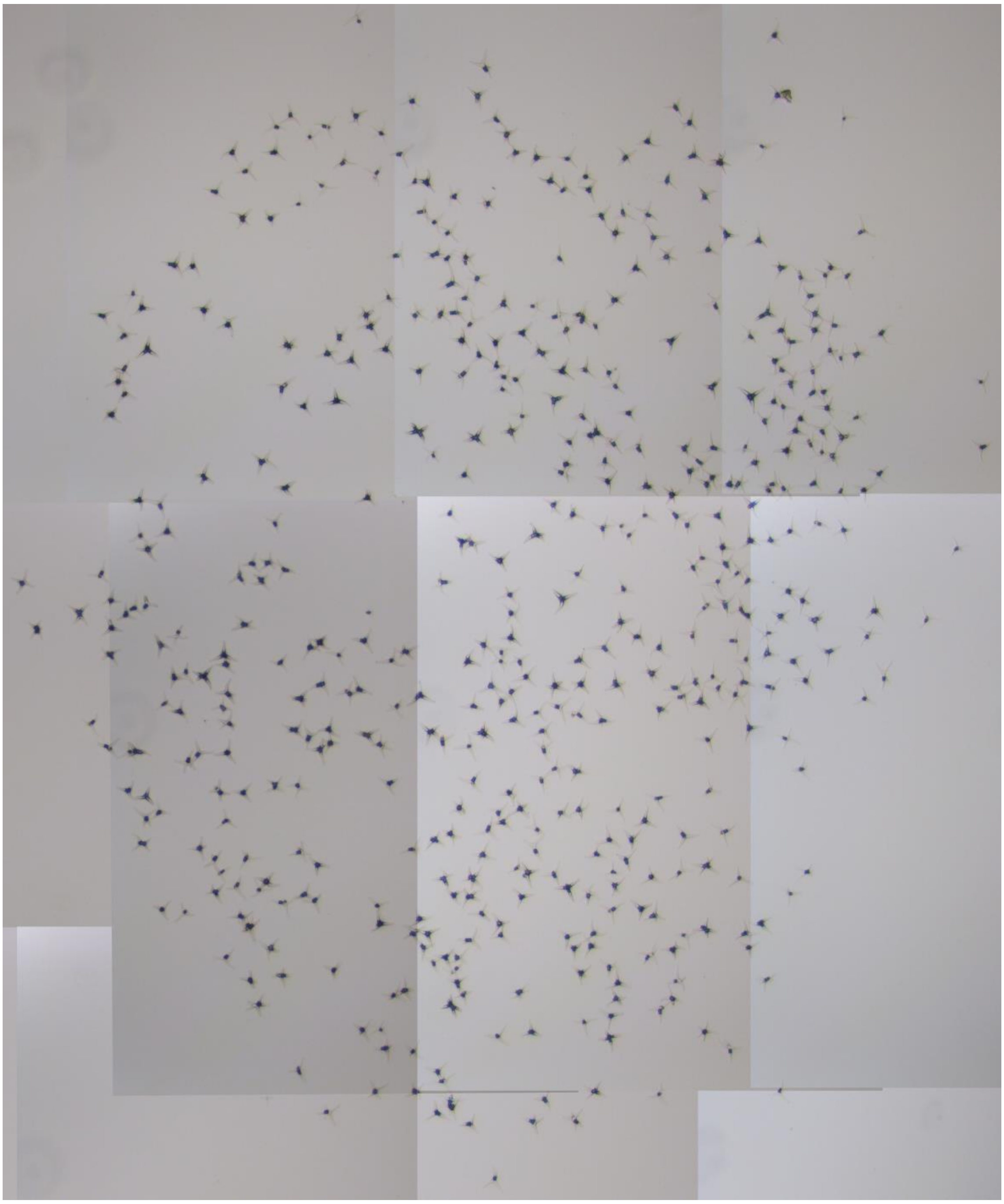

Fig. S2. Isolated *ca.* 500 cells of *Chionaster nivalis* from the green snow sample of Oze National Park (Fig. 1c), shown as a composite micrograph. The cells were assigned to the specimen Oze and used for molecular analysis.

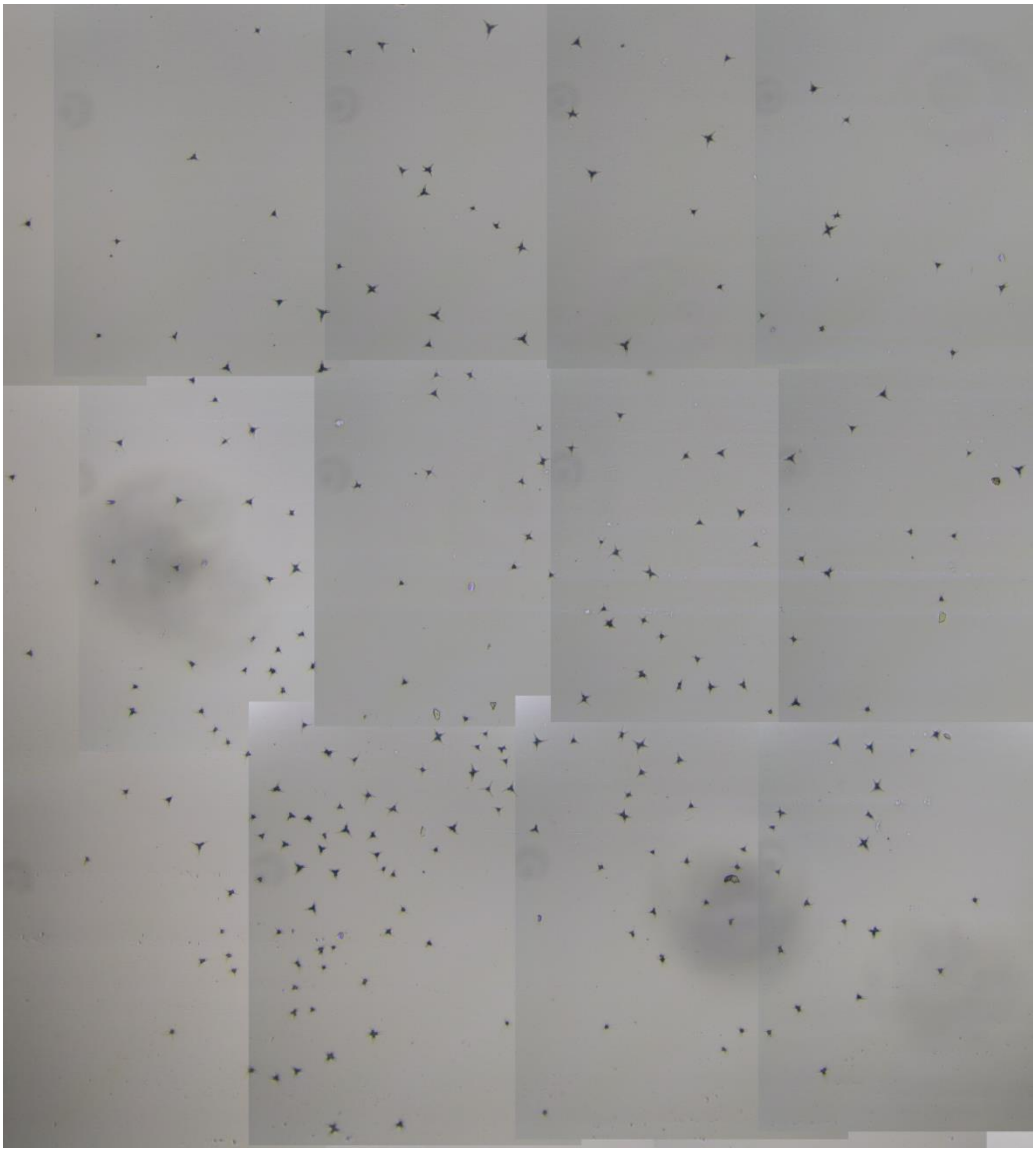

Fig. S3. Isolated *ca.* 200 cells of *Chionaster nivalis* from the brown snow sample of Tambara-kogen Plateau (Fig. 1d), shown as a composite micrograph. The cells were assigned to the specimen Tambara and used for molecular analysis.

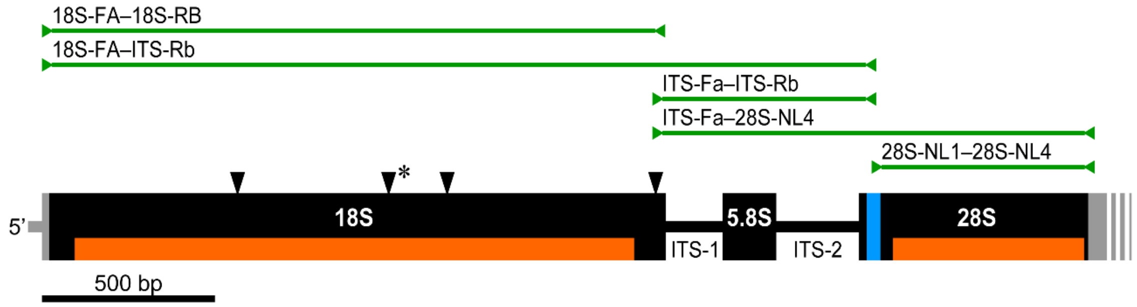

Fig. S4. Schematic diagram of the Sanger-sequenced nuclear ribosomal RNA operon of *Chionaster nivalis* specimens Oze and Tambara. Abbreviation: ITS, internal transcribed spacer. Black boxes and lines show the regions sequenced in this study. Note that a part of the 28S ribosomal DNA in the specimen Oze (shown in blue) was not sequenced. The amplified regions and primer set in the respective polymerase chain reactions are shown above the diagram (see also Method S3 and Table S1). Arrowheads on the 18S ribosomal RNA exhibit the positions in which group I introns are inserted. The asterisk means that the intron was observed only in the specimen Oze. Red bars indicate the regions used for molecular phylogenetic analyses (Fig. 2).

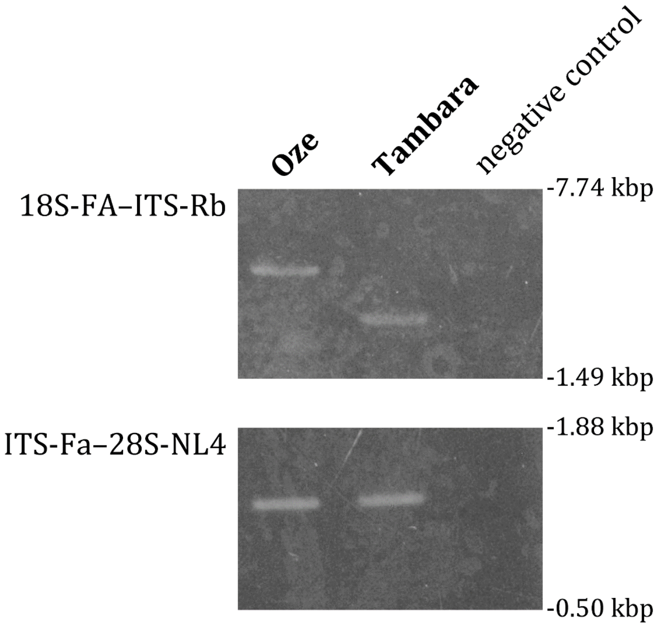

Fig. S5. Results of the PCR for the DNA samples obtained from more than 200 cells of *Chionaster nivalis*. For information of primers, see Table S1 and Fig. S4. Note that the number of group I intron inserted in 18S ribosomal DNA was different between the specimens Oze and Tambara (see Fig. S4).

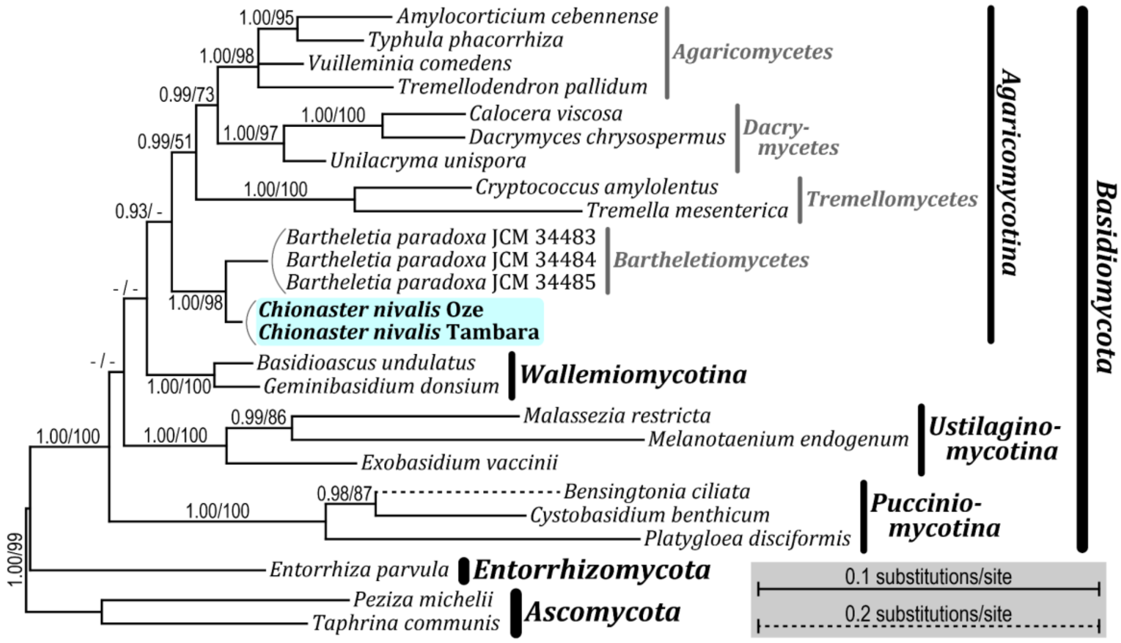

Fig. S6. Bayesian phylogenetic tree of *Basidiomycota* based on 18S ribosomal RNA sequences. Names of classes, subphyla, and phyla are according to the previous study by Naranjo-Ortiz and Gabaldón (2019). The corresponding posterior probabilities of Bayesian inference (0.90 or more, left) and bootstrap values from maximum likelihood analysis (50% or more, right) are shown at each node.

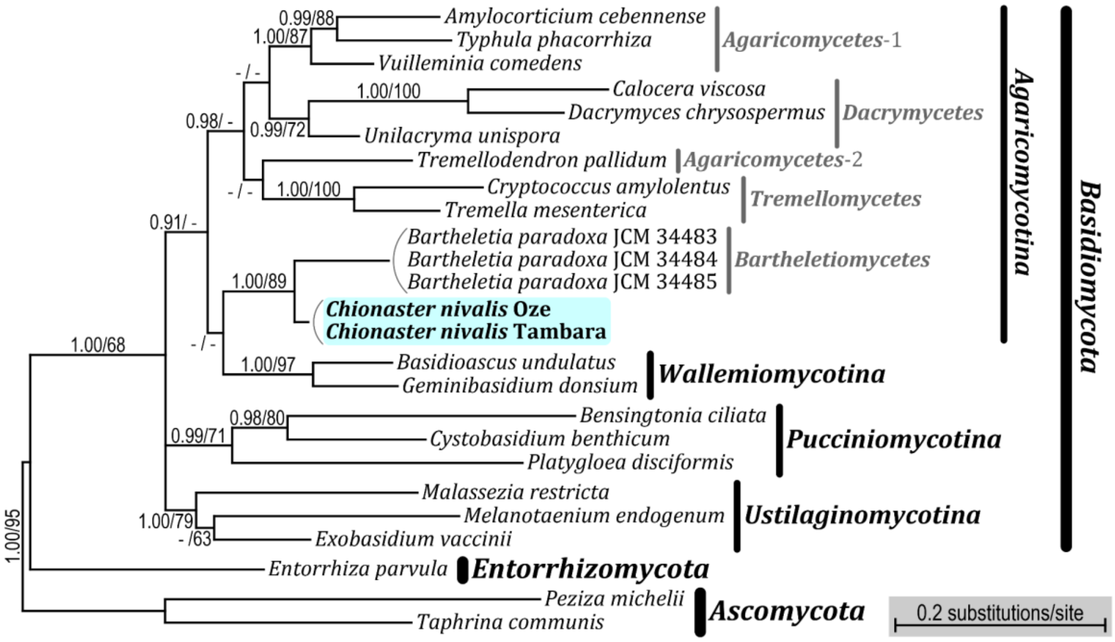

Fig. S7. Bayesian phylogenetic tree of *Basidiomycota* based on 28S ribosomal RNA sequences. Names of classes, subphyla, and phyla are according to the previous study by Naranjo-Ortiz and Gabaldón (2019). The corresponding posterior probabilities of Bayesian inference (0.90 or more, left) and bootstrap values from maximum likelihood analysis (50% or more, right) are shown at each node.

Supplementary References

Binder, M., Larsson, K.H., Matheny, P.B., and Hibbett, D.S. (2010) *Amylocorticiales* ord. nov. and *Jaapiales* ord. nov.: Early diverging clades of *Agaricomycetidae* dominated by corticioid forms. *Mycologia* **102**: 865–880.

Capella-Gutiérrez, S., Silla-Martínez, J.M., and Gabaldón, T. (2009) TrimAl: A tool for automated alignment trimming in large-scale phylogenetic analyses. *Bioinformatics* **25**: 1972–1973.

Coleman, A.W., Suarez, A., and Goff, L.J. (1994) Molecular delineation of species and syngens in volvocacean green algae (*Chlorophyta*). *J Phycol* **30**: 80–90.

Darriba, D., Posada, D., Kozlov, A.M., Stamatakis, A., Morel, B., and Flouri, T. (2020) ModelTest-NG: A new and scalable tool for the selection of DNA and protein evolutionary models. *Mol Biol Evol* **37**: 291–294.

Fan, M., Currie, B.P., Gutell, R.R., Ragan, M.A., and Casadevall, A. (1994) The 16S-like, 5.8S and 23S-like rRNAs of the two varieties of *Cryptococcus neoformans*: sequence, secondary structure, phylogenetic analysis and restriction fragment polymorphisms. *J Med Vet Mycol* **32**: 163–180.

Felsenstein, J. (1985) Confidence limits on phylogenies: An approach using the bootstrap. *Evolution* **39**: 783–791.

Hall, T.A. (1999) BioEdit: A User-friendly biological sequence alignment editor and analysis program for Windows 95/98/NT. *Nucleic Acids Symp Ser* **41**: 95–98.

Kasai, F., Kawachi, M., Erata, M., Mori, F., Yumoto, K., Sato, M., and Ishimoto, M. (2009) NIES-collection. List of strains, ed. 8. *Japanese Journal of Phycology (Sôrui)* **57**(1) supplement: 1–350, pls 1–7.

Kato, S. (1982) Laboratory culture and morphology of *Colacium vesiculosum* Ehrb. (*Euglenophyceae*). *Japanese Journal of Phycology (Sôrui)* **30**: 63–67 (in Japanese with English abstract).

Katoh, K., and Standley, D.M. (2013) MAFFT multiple sequence alignment software version 7: Improvements in performance and usability. *Mol Biol Evol* **30**: 772–780.

Kawai-Toyooka, H., Kuramoto, C., Orui, K., Motoyama, K., Kikuchi, K., Kanegae, T., and Wada, M. (2004) DNA interference: A simple and efficient gene-silencing system for high-throughput functional analysis in the fern *Adiantum*. *Plant Cell Physiol* **45**: 1648–1657.

Kirk, D., and Kirk, M.M. (1983) Protein synthetic patterns during the asexual life cycle of *Volvox carteri*. *Dev Biol* **96**: 493–506.

Koukol, O., and Lotz-Winter, H. (2016) Secondary conidia observed in *Bartheletia paradoxa*. *Czech Mycol* **68**: 79–84.

Kozlov, A.M., Darriba, D., Flouri, T., Moreil, B., and Stamatakis, A. (2019) RAxML-NG: A fast, scalable, and user-friendly tool for maximum likelihood phylogenetic inference. *Bioinformatics* **35**: 4453–4455.

Kurtzman, C.P., and Robnett, C.J. (1997) Identification of clinically important ascomycetous yeasts based on nucleotide divergence in the 5' end of the large-subunit (26S) ribosomal DNA gene. *J Clin Microbiol* **35**: 1216–1223.

Millanes, A.M., Diederich, P., Ekman, S., and Wedin, M. (2011) Phylogeny and character evolution in the jelly fungi (*Tremellomycetes*, *Basidiomycota*, Fungi). *Mol Phylogenet Evol* **61**: 12–28.

Miura, K., and Kudo, M. (1970) An ager-medium for aquatic hyphomycetes. *Trans Mycol Soc Japan* **11**: 116–118 (in Japanese).

Muramoto, K., Nakada, T., Shitara, T., Hara, Y., and Nozaki, H. (2010) Re-examination of the snow algal species *Chloromonas miwae* (Fukushima) Muramoto *et al.* comb., nov. (*Volvocales*, *Chlorophyceae*) from Japan, based on molecular phylogeny and cultured material. *Eur J Phycol* **45**: 27–37.

Nakada, T., Suda, S., and Nozaki, H. (2007) A taxonomic study of *Hafniomonas* (*Chlorophyceae*) based on a comparative examination of cultured material. *J Phycol* **43**: 397–411.

Nakazawa, A., and Nozaki, H. (2004) Phylogenetic analysis of the tetrasporalean genus *Asterococcus* (*Chlorophyceae*) based on 18S ribosomal RNA gene sequences. *Journal of Japanese Botany* **79**: 255–261.

Naranjo-Ortiz, M.A., and Gabaldón, T. (2019) Fungal evolution: Diversity, taxonomy and phylogeny of the Fungi. *Biol Rev Camb Philos Soc* **94**: 2101–2137.

Nozaki, H., Watanabe, M.M., and Aizawa, K. (1995) Morphology and paedogamous sexual reproduction in *Chlorogonium capillatum* sp. nov. (*Volvocales*, *Chlorophyta*). *J Phycol* **31**: 655–663.

Nozaki, H., Nakada, T., and Watanabe, S. (2010) Evolutionary origin of *Gloeomonas*

(*Volvocales*, *Chlorophyceae*), based on ultrastructure of chloroplasts and molecular phylogeny. *J Phycol* **46**: 195–201.
Nozaki, H., Ueki, N., Misumi, O., Yamamoto, K., Yamashita, S., Herron, M.D., and Rosenzweig, F. (2015) Morphology and reproduction of *Volvox capensis* (*Volvocales*, *Chlorophyceae*) from Montana, USA. *Phycologia* **54**: 316–320.
Pringsheim, E.G. (1946) *Pure cultures of algae*. London, UK: Cambridge University Press.
Rognes, T., Flouri, T., Nichols, B., Quince, C., and Mahé, F. (2016) Vsearch: A versatile open source tool for metagenomics. *PeerJ* **4**: e2584.
Ronquist, F., Teslenko, M., van der Mark, P., Ayres, D.L., Darling, A., Höhna, S., *et al.* (2012) MrBayes 3.2: Efficient Bayesian phylogenetic inference and model choice across a large model space. *Syst Biol* **61**: 539–542.
Sugasawa, M., Matsuzaki, R., Kawafune, K., Takahashi, T., Kawachi, M., Krienitz, L., and Nozaki, H. (2015) Taxonomic study of *Pyrobotrys* (*Spondylomoraceae*, *Chlorophyceae*) based on comparative morphological and molecular analyses of culture strains established using novel methods. *Cytologia* **80**: 513–524.
Takashima, Y., Seto, K., Degawa, Y., Guo, Y., Nishizawa, T., Ohta, H., and Narisawa, K. (2018) Prevalence and intra-family phylogenetic divergence of *Burkholderiaceae*-related endobacteria associated with species of *Mortiella*. *Microbes Environ* **33**: 417–427.
Vilgalys, R., and Hester, M. (1990) Rapid genetic identification and mapping of enzymatically amplified ribosomal DNA from several *Cryptococcus* species. *J Bacteriol* **172**: 4238–4246.
White, T.J., Bruns, T., Lee, S., and Taylor, J.W. (1990) Amplification and direct sequencing of fungal ribosomal RNA genes or phylogenetics. In *PCR Protocols: A Guide to Methods and Applications*. Innis, M.A., Gelfand, D.H., Sninsky, J.J., White, T.J. (eds). New York, USA: Academic Press, pp. 315–322.
Zhao, R-L., Li, G-J., Sánchez-Ramírez, S., Stata, M., Yang, Z-L., Wu, G., *et al.* (2017) A six-gene phylogenetic overview of *Basidiomycota* and allied phyla with estimated divergence times of higher taxa and a phyloproteomics perspective. *Fungal Divers* **84**: 43–74.
